# The Roller Coaster of Lamotrigine Levels: Successful Treatment of Massive Lamotrigine Overdose With Continuous Veno-Venous Hemodiafiltration and Rifampin

**DOI:** 10.7759/cureus.65637

**Published:** 2024-07-29

**Authors:** Yi Li, Hans S Ang, Pedram Fatehi, Natalie Htet

**Affiliations:** 1 Critical Care, Stanford University School of Medicine, Stanford, USA; 2 Pharmacy, Stanford University Medical Center, Stanford, USA; 3 Nephrology, Stanford University, Stanford, USA; 4 Emergency Medicine, Stanford University, Stanford, USA

**Keywords:** hemodialysis, lipid emulsion, rifampin, continuous hemodiafiltration, lamotrigine overdose

## Abstract

Lamotrigine is a commonly used anticonvulsant in treating seizures and bipolar disorder, but there is very limited literature on the management of its toxicity. Case reports have been published suggesting the potential role of hemodialysis in lowering serum lamotrigine levels, as well as sodium bicarbonate and lipid emulsion in treating dysrhythmia. After previously reported therapies failed to stabilize the patient's condition, the case presents our successful treatment experience using continuous veno-venous hemodiafiltration (CVVHDF) to stabilize lamotrigine levels, as well as intravenous rifampin as adjunctive therapy to facilitate lamotrigine metabolism. This is a 66-year-old male who was found unresponsive after a lamotrigine overdose. His first lamotrigine level was 42.3 ug/mL. Hemodialysis was started on hospital day 1. Despite hemodialysis sessions, his lamotrigine level rebounded with worsening neurological and cardiac symptoms. On hospital day 3, he developed wide-QRS complex tachyarrhythmia and hemodynamic instability with a lamotrigine level of 66.9 ug/mL. Sodium bicarbonate was given without effect. Lipid emulsion was administered which terminated the arrhythmia. CVVHDF and rifampin were started and lamotrigine levels have continuously downtrended since. He was successfully extubated on day 7. Lamotrigine level became undetectable on day 9. The patient was discharged to a psychiatric facility without any neurological or mobility impairment on day 10. The continuous drug clearance provided by CVVHDF over intermittent hemodialysis may have provided additional benefit in lamotrigine level stabilization, while rifampin use in this case may have further accelerated lamotrigine metabolism. As the first case reporting CVVHDF and rifampin use, our experience suggests their potential roles in managing severe lamotrigine toxicity.

## Introduction

Lamotrigine is a commonly used anticonvulsant in treating seizures and bipolar disorder. No antidote is available and there is limited data on managing lamotrigine overdose. Conventional treatment is largely symptomatic and supportive awaiting lamotrigine metabolism and excretion. Existing case reports suggest that hemodialysis can increase lamotrigine drug clearance and positive outcomes were achieved [1,2]. Sodium bicarbonate and lipid emulsion have been used in treating wide-QRS complex dysrhythmia with various success [3]. This report presents a case of massive lamotrigine overdose with significant level rebound phenomenon despite hemodialysis, as well as successful management with continuous veno-venous hemodiafiltration (CVVHDF) and intravenous rifampin resulting in excellent outcomes.

## Case presentation

**Figure 1:**
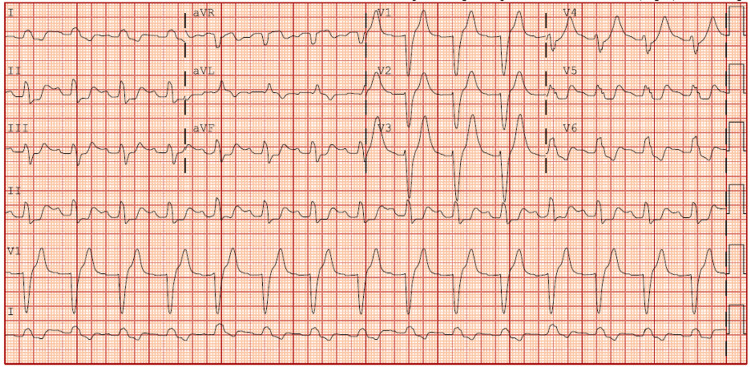
The new wide-QRS complex arrhythmia that developed in the early morning of hospital day 3, when the patient’s lamotrigine level peaked.

**Figure 2:**
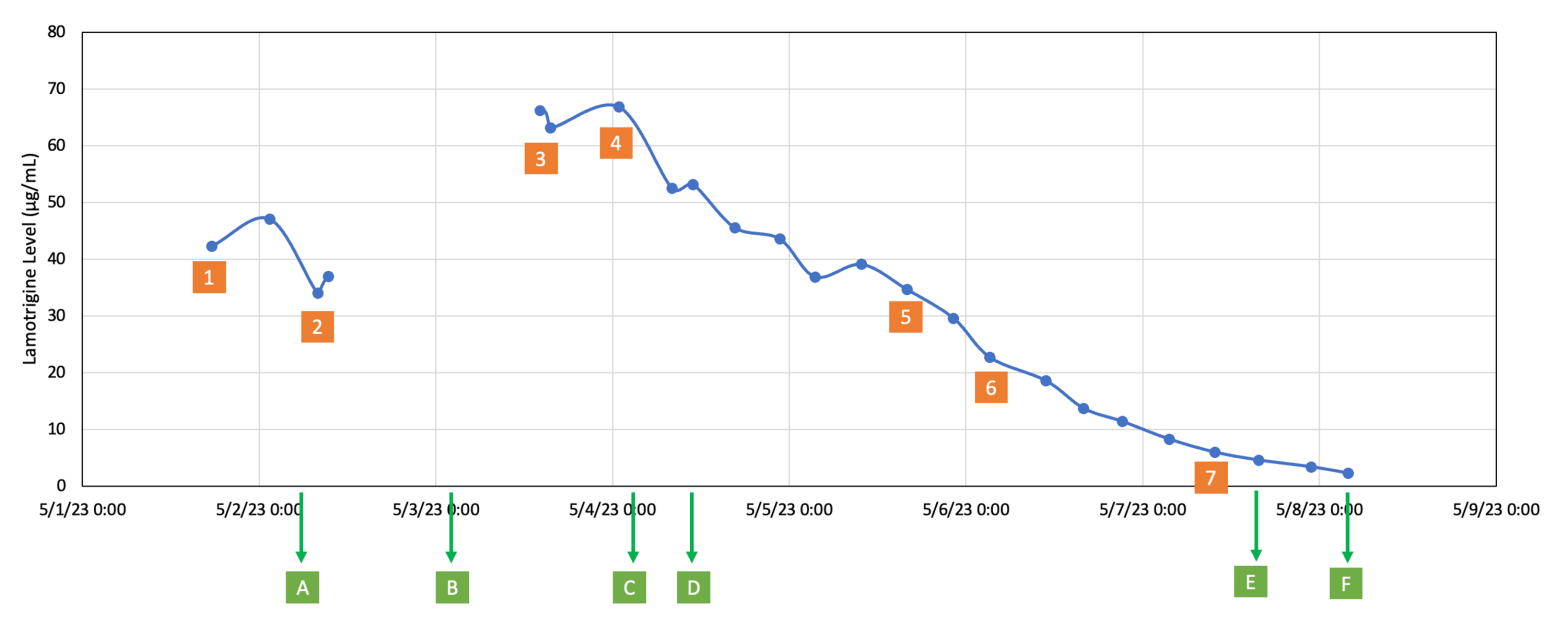
Timeline of lamotrigine levels, neurological and cardiac symptoms, and treatment Numbers 1 to 7 represent the patient’s neurological and cardiac symptoms: 1. Unresponsive, Glasgow Coma Scale (GCS) of 6. 2. Awake and able to follow commands. 3. Flexion of upper extremities and trace movement of lower extremities with stimulation. 4. Trace movement of upper extremities and no movement of lower extremities with stimulation, accompanied by wide-QRS complex tachyarrhythmia and hemodynamic instability. 5. Arousable to voice with spontaneous movement of extremities but not following commands. 6. Awake and able to follow commands. 7. Awake, alert, oriented to four, and able to follow commands consistently. Letters A to F represent management interventions: A. First hemodialysis session. B. Second hemodialysis session. C. Sodium bicarbonate and lipid emulsion administered, and continuous veno-venous hemodiafiltration (CVVHDF) started. D. Rifampin started. E. Extubated, CVVHDF discontinued, heparin bolus and thrombectomy for deep vein thrombosis (DVT). F. Rifampin discontinued. Note: The line before the fourth and fifth lamotrigine levels was removed to prevent any misunderstanding that the second dialysis session might have elevated lamotrigine levels.

A 66-year-old male (86 kg, 188 cm, body mass index 24.3 kg/m^2^), with a history of chronic kidney disease stage 3, bladder cancer status post cystectomy and ileal conduit in remission, left-sided neobladder stricture with nephrostomy tube in place, and bipolar disorder, was found unresponsive. An empty bottle of lamotrigine and a suicide note was found next to him. The patient was presumed to have taken 21 g of lamotrigine in the form of immediate-release tablets six hours prior to arrival. His vital signs were blood pressure of 104/70 mmHg, heart rate of 48/min, temperature of 35.0℃, respiratory rate of 20/min, and pulse oximetry of 100% on room air. His Glasgow Coma Scale was 6 and was intubated for airway protection. Admission laboratory tests were overall unremarkable except for slightly elevated serum creatinine of 1.34 mg/dL with an estimated glomerular filtration rate (eGFR) of 59 ml/min/1.73m^2^, both of which were at his baseline (Table 1). His computed tomography (CT) imaging of the head, chest, abdomen, and pelvis was unremarkable and negative for radiographic evidence of bezoar formation. His continuous electroencephalogram (EEG) was negative for seizure. The first lamotrigine level was 42.3 ug/mL and the second was 47.1 ug/mL. The patient was started on hemodialysis on hospital day 1. After the first session of dialysis (using a high-flux dialyzer, with a dialysate flow rate of 600 mL/min, a blood flow rate of 350 mL/min, and a duration of 3.5 hours), the lamotrigine level decreased to 34.0 ug/mL immediately post-dialysis and his neurological exam improved to be able to follow simple commands. Two hours after dialysis, the repeat lamotrigine level was 37.0 ug/mL. A second session of hemodialysis was performed on hospital day 2 (using a high-flux dialyzer with a dialysate flow rate of 600 mL/min, a blood flow rate of 350 mL/min, and a duration of 3.5 hours). Despite the second session, his post-dialysis lamotrigine level was 66.2 ug/mL. His neurological exam worsened to flexion of upper extremities and trace movement of lower extremities. He then developed wide-QRS complex tachyarrhythmia with a new left bundle branch block pattern (Figure 1) and became hemodynamically unstable requiring norepinephrine and vasopressin to maintain mean arterial pressure (MAP) above 65 mmHg. A total of 400 mEq of sodium bicarbonate was administered in both bolus and infusion form to the effect of pH 7.58 without any rhythm change. A total of 1.5 mL/kg of lipid emulsion intravenous bolus was given, followed by 0.25 mL/kg/min intravenous infusion for one hour. The rhythm then returned to normal sinus rhythm shortly after. However, his neurological exam worsened to only trace movement of the upper extremities and no movement of the lower extremities despite stimulation. His lamotrigine level drawn during the arrhythmic episode was 66.9 ug/mL. He was started on CVVHDF (blood flow 200-250 mL/min, pre-replacement 600 mL/hour with PrismaSol B22GK4/0 bicarb 22 (Baxter International Inc., Deerfield, USA), post-replacement 400-850 mL/hour with PrismaSol B22GK4/0 bicarb 22, dialysate flow 600-850 mL/hour with PrismaSol B22GK4/0 bicarb 22). Regional citrate anticoagulation was used in CVVHDF. After 4.5 hours of CVVHDF initiation, the lamotrigine level decreased to 52.5 ug/mL. On day 3, we started intravenous rifampin at 300 mg every eight hours based on literature indicating rifampin can potentially increase lamotrigine clearance [4]. On hospital days 4 to 5, his lamotrigine level continued to a downtrend and his neurological exam improved gradually to arousable to voice with spontaneous movement of extremities but not following commands. On hospital day 6, he started following commands with a lamotrigine level of 22.7 ug/mL. On hospital day 7, lamotrigine level was 6.0 ug/mL, and he was successfully extubated. Due to the high-pressure alarm, CVVHDF was stopped, and the temporary dialysis catheter was removed from the right femoral vein. Despite the prophylactic subcutaneous heparin use since admission, the patient developed phlegmasia cerulea dolens of the right leg after dialysis catheter removal. His CT venogram showed new occlusive deep venous thrombosis (DVT) involving the entirety of the right external iliac, proximal right internal iliac, and common femoral veins. He was given heparin intravenous bolus of 80 units/kg and underwent a successful thrombectomy. Subcutaneous enoxaparin was started at 1 mg/kg twice a day. Rifampin was discontinued on hospital day 8. Lamotrigine level became undetectable on hospital day 9. The patient was discharged to a psychiatric facility on hospital day 10 without any neurological impairment (Figure 2).

**Table 1:** The venous blood laboratory test results on admission showed no significant abnormality except for slightly elevated renal function which is at the patient's baseline due to a history of chronic kidney disease.

Laboratory test	Value	Normal range
White blood cell count (K/mm^3^)	9.5	3.5-10.8
Hemoglobin level (g/dL)	12.6	13.5-17.5
Platelet count (K/mm^3^)	206	150-400
Serum sodium level (mEq/L)	138	136-144
Serum sodium level (mEq/L)	3.9	3.7-5.2
Serum bicarbonate level (mmol/L)	21	23-29
Serum creatinine level (mg/dL)	1.34	0.8-1.2
Estimated glomerular filtration rate (ml/min/1.73 m^2^)	59	≥60
Blood urea nitrogen (mg/dL)	23	6-20
Aspartate aminotransferase (IU/L)	18	10-40
Alanine aminotransferase (IU/L)	15	10-40

## Discussion

Lamotrigine is a commonly used anticonvulsant in treating epilepsy and bipolar disorder. It inhibits the release of glutamate and inhibits voltage-sensitive sodium channels to reduce repetitive neuronal firing. However, the shared homology of sodium channels and lack of selectivity can cause slowing cardiac conduction and increased proarrhythmic potential [5]. Despite being anticonvulsant, lamotrigine toxicity can cause central nervous system dysfunction, manifesting variously from coma and encephalopathy, to seizure. Oral lamotrigine is rapidly absorbed with an absolute bioavailability of 98% with time to peak plasma concentration ranging between 2.2 and 3 hours and a half-life between 25.4 and 32.8 hours, longer in patients with renal dysfunction [6]. Lamotrigine is metabolized in the liver primarily by glucuronic acid conjugation into inactive metabolites and eliminated in urine as its corresponding 2N-glucuronic acid conjugate [7,8]. In this case, the lamotrigine levels correlated well with the degree of cardiac and neurological symptoms. However, the prolonged turnaround time for lamotrigine levels (8-24 hours) made it difficult to use lamotrigine levels to guide real-time management.

There is a limited number of literature discussing lamotrigine toxicity management, especially in adults. The main source of evidence comes from case reports. Toxicity interventions include supportive care, sodium bicarbonate, lipid emulsion, and extracorporeal elimination through renal replacement therapy [1,2]. Although lamotrigine is only partially dialyzable due to its serum protein binding of 55% [9], a human study by Fillastre et al. showed that hemodialysis shortened the elimination half-life of lamotrigine from 59.6 ± 28.1 hours to 12.2 ± 6.4 hours, and 17% extraction of lamotrigine dose was observed through hemodialysis [6]. The role of lipid emulsion has been reported in recovering normal cardiac conduction [10], especially in cases where sodium bicarbonate was not breaking cardiac arrhythmia. Our experience was similar, and we think caution should be placed about relying solely on sodium bicarbonate.

In this case, hemodialysis was initiated due to the severity of symptoms and previously reported experience of success. After the first 3.5-hour session of hemodialysis, the lamotrigine level decreased from 47.1 ug/mL to 34.0 ug/mL, representing an elimination rate of 27.8%. This is consistent with existing literature reporting a 20% elimination rate. However, a post-hemodialysis rebound phenomenon, which was never reported before, was observed. Despite a second session of hemodialysis, the patient's lamotrigine level rebounded to 66.2 ug/mL, associated with worsening symptoms. Unfortunately, no lamotrigine level was collected immediately prior to the second session, so we are unable to assess if the level of 66.2 ug/mL represents an increase or decrease after the second session. Our assumption is it likely represents a decrease due to the eliminating effect already demonstrated by the first session. The reason for the rebound remains unknown. To our knowledge, there was no report on bezoar formation by lamotrigine tablets and this patient’s imaging did not show any evidence of bezoar formation. Ongoing absorption is unlikely, because the peak level of lamotrigine, in this case, occurred approximately 48 hours after ingestion, which is much longer than previously reported time to peak concentration for either immediate release (1-5 hours) or extended-release (4-11 hours) formation [11], and there was no reason to believe patient, in this case, would have delayed absorption compared to the general population. Another possibility is peri-hemodialysis drug redistribution as it was only until CVVHDF was started that the lamotrigine levels started consistently downtrending. Although there is no report on CVVHDF use in treating lamotrigine toxicity, continuous renal replacement therapy (CRRT) has been used in treating severe lithium toxicity for the risk of post-dialysis rebound concentration [12,13]. Together with the unstable lamotrigine levels within 48 hours of ingestion in this case, the experience suggests a potential beneficial role of CVVHDF in stabilizing lamotrigine levels, preventing rebound phenomenon, and thus preventing cardiac and neurological instability. One could argue a prolonged or even non-stop hemodialysis sessions could have achieved similar or even better stabilization due to a higher clearance rate. This is a valid point and can be attempted. Our experience only represents a successful real-world case using CVVHDF and the emphasis is primarily on continuous instead of intermittent clearance. Logistical and personnel factors should be taken into consideration if a prolonged or non-stop hemodialysis session is performed.

**Figure 3:**
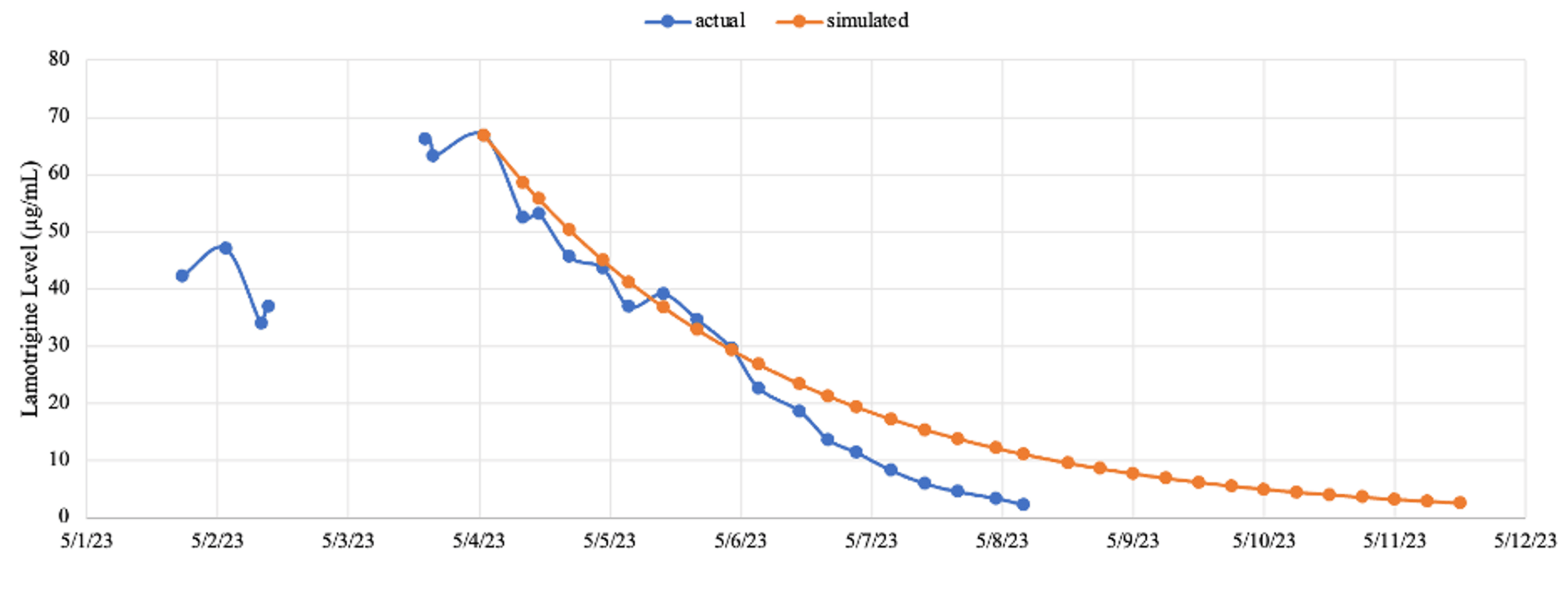
Comparison of actual versus simulated lamotrigine levels The simulated lamotrigine levels were produced using the actual observed elimination rate of lamotrigine (with continuous veno-venous hemodiafiltration) prior to rifampin initiation. The first-order kinetic equation was applied. A faster elimination effect of lamotrigine was observed with rifampin use compared to the simulated levels trend.

Rifampin was used based on the drug-drug interaction between rifampin and lamotrigine [4]. In an experimental study in healthy male humans, rifampin co-administration significantly increased the amount of lamotrigine excreted as glucuronide by 37% and decreased terminal half-life by 40%. Such effect was believed due to the induction of hepatic enzymes responsible for glucuronidation, which takes place 24 hours after rifampin initiation and reaches 90% maximal induction between days 5 and 9 [14]. The lamotrigine levels and the change of elimination half-life in this case largely correlate with this timeline (Table 2). Although it is impossible to delineate the exact role rifampin played due to the concurrent use of CVVHDF, we compared the actual lamotrigine level trend with a simulated level trend using the eliminate rate before rifampin initiation. A discrepancy was observed, especially after 24 hours of rifampin initiation (Figure 3). Such discrepancy suggests that rifampin likely increased the elimination of lamotrigine in this case. Given the low-risk side effect profile of rifampin, we believe rifampin can be considered as an adjunctive therapy for lamotrigine toxicity.

**Table 2:** Actual lamotrigine levels and the elimination half-life change after rifampin initiation This table illustrates the changes in lamotrigine levels as well as the elimination of half-life using the first-order kinetic equation before and after rifampin initiation. Rifampin was started on day 3 after lamotrigine level 52.5 resulted. The elimination half-life shortened significantly after rifampin initiation. “D” is the abbreviation of hospital day.

Level 1 time	Level 1 (ug/mL)	Level 2 time	Level 2 (ug/mL)	Interval (hour)	Elimination half-life between levels 1 and 2 (hour)
D3 8:06	52.5	D4 3:32	36.9	19.4	35.2
D4 3:32	36.9	D5 3:15	22.7	23.7	31.2
D5 3:15	22.7	D6 3:35	8.3	24.3	15.5
D6 3:35	8.3	D7 3:57	2.3	24.4	12.1

## Conclusions

The successful experience in this case suggests two novel strategies for managing severe lamotrigine toxicity. First is the potential advantage of CRRT over intermittent hemodialysis. Despite the lower clearance rate, the continuous clearance nature may have provided steady drug clearance during drug redistribution and therefore prevented level spikes and rebound. Such an advantage can be critical in treating lamotrigine toxicity given the close correlation between lamotrigine levels and severity of symptoms, especially cardiovascular symptoms. Second, intravenous rifampin likely increased lamotrigine elimination in this case and can be considered as a safe adjunctive therapy if there is no contraindication, although its exact effect on lamotrigine levels is unable to be fully determined. Sodium bicarbonate alone did not demonstrate success in converting wide-QRS complex arrhythmia in our experience and clinicians should remain cautious in relying solely on it. Lipid emulsion did demonstrate success and should be considered when treating arrhythmia caused by lamotrigine toxicity. Given the limitations of our report, future studies focusing on lamotrigine level stabilization may help confirm the advantage of CRRT in treating severe lamotrigine toxicity. Future reports on rifampin use may provide additional evidence supporting its role in treating lamotrigine toxicity.
